# A non-linear flow model for the flow behavior of water inrush induced by the karst collapse column

**DOI:** 10.1039/c7ra11344g

**Published:** 2018-01-05

**Authors:** Xian'gang Hou, Wenhao Shi, Tianhong Yang

**Affiliations:** Center of Rock Instability and Seismicity Research, School of Resources and Civil Engineering, Northeastern University Shenyang 110819 China xianganghou@126.com shiwenhaoneu@126.com +86-24-8367-1626; Key Laboratory of Ministry of Education on Safe Mining of Deep Metal Mines, Northeastern University Shenyang 110819 China yangtianhong@mail.neu.edu.cn

## Abstract

Water inrush induced by the karst collapse column (KCC) is a great threat to coal mine safety. In this study, a non-linear flow model that couples three flow types is built based on flow transition from laminar flow in the aquifer to turbulent flow in the mine roadways during the process of water inrush induced by KCC. The proposed model couples Darcy flow, Forchheimer flow, and turbulent flow, and is then used to simulate the flow behavior of water inrush induced by KCC. In particular, the “3.1” water inrush incident from the coal seam floor in the Luotuoshan coal mine, China, is numerically investigated. The numerical results show that with the increase of the inrush flow rate, Forchheimer flow in the water inrush channel is first controlled by viscous resistance, then affected by both viscous resistance and inertial resistance, and finally controlled by inertial resistance. Therefore, water inrush induced by KCC is a dynamic process with a transition from laminar to turbulent. The Forchheimer equation proved to be applicable in describing the high-velocity non-linear flow, and can also reflect the intermediate state of the flow translation from laminar flow in the aquifer to turbulent flow in the roadway during the water inrush process.

## Abstract 2

A non-linear flow model that couples three flow types is built based on flow transition to investigate the flow behavior of water inrush induced by KCC.

## Graphical abstract

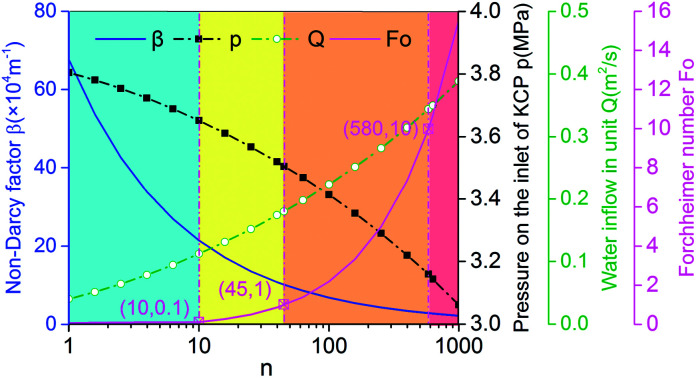

## 1. Introduction

The karst collapse column (KCC) is one special type of karst collapse in the permo-carboniferous coal field of North China. The KCC is composed of a fractured rock mass of coal measure strata. Water inrush induced by KCC can be sudden and catastrophic,^1,2^ which threatens the safety of coal mines. KCCs are widely dispersed in almost 45 coal mines attached to 20 coal fields in Shanxi, Shandong, Henan, Jiangsu, Hebei, and Shaanxi provinces of China.^2,3^ Many Chinese coal seams lie above the carbonate karst rocks. The total number of Chinese coal mines has increased to over 3000.^4^ Long tunnel construction projects are also threatened by karst water inrush. According to the relevant statistical data, large-scale karst water inrushes have taken place in almost 50% of the 17 karst tunnels located in southwest and middle-south China since 1989.^5^

Water inrush induced by KCC indicates that the rock mass in the KCC has hydraulically connected a karst aquifer and the mine roadway. The relationship between the velocity and pressure gradient of the inrush in a fractured rock mass generally does not satisfy the Darcy equation, and is distinctly nonlinear.^6–10^ It is difficult to describe the non-linear flow pattern based on the Darcy equation, which is applicable in linear flow only. Therefore, it is of great theoretical and practical significance to build a nonlinear flow model for the identification of flow mechanisms and the reasonable forecast of water inrush.

Two main equations are used to describe the non-linear relationship between the pressure and flow rate of fluids in porous media: the Izbash equation and the Forchheimer equation (see f.e.).^11,12^ The Forchheimer equation was first proposed based on experiment and then later demonstrated by theoretical inference.^13,14^ The first-degree term of the Forchheimer equation is associated with viscous resistance, and the quadratic term is associated with inertial resistance. When the flow rate is small enough, the viscous resistance is the main factor affecting the flow behavior. However, when the flow rate is high, inertial resistance is the main factor. Compared with the Izbash equation, the Forchheimer equation has clear theoretical and physical background, which can describe the high-velocity non-linear flow in porous media (with large porosity) and in fractured media.^15–18^

Water inrush induced by KCC in mining engineering shows the characteristics of Forchheimer flow. Miao *et al.*^19^ and Ma *et al.*^6^ conducted seepage experiments on broken rocks and found that the seepage in the broken rock obeys the Forchheimer equation. Sedghi-Asl *et al.*^20^ conducted seepage experiments on different aggregate size gravels and found that the non-Darcy factor decreases as the aggregate size increases based on the Forchheimer equation. Different types of porous media were also examined to experimentally study the parameters of the Forchheimer equation and these results were systematically summarized by Moutsopoulos *et al.*^21^

In recent years, numerical simulation has been widely used to study high-velocity non-Darcy flow behavior in sand and gravel riverbeds,^22^ rockfills,^23^ and earth and rockfill dams.^24^ Basak *et al.*^25^ presented the effect of non-linearity in the flow response on the discharge characteristics and pressure distribution of a non-penetrating well in a semi-infinite medium incorporating the Forchheimer equation. Wang *et al.*^26^ also performed similar studies. These models are all based on the Forchheimer equation. Moreover, Xu *et al.*^27,28^ proposed an innovative simulation method to study the flow state evolution laws in the karst regions by coupling Darcy's Law, Brinkman equations and the incompressible Navier–Stokes equations.

Groundwater travels from an aquifer though a KCC into a mine roadway during the process of water inrush induced by the KCC. The history of the pressure and velocity of each flow field is a time-varying physical process and hence, the three flow fields are inseparable both in time and space.^29^ Although laboratory tests^20,22^ and theoretical analysis^13,22,30^ are precise and reliable in revealing the non-linear flow mechanisms of Forchheimer flow in fractured rock masses, the flow mechanisms of Forchheimer flow for large-scale engineering problems still cannot be quantitatively analyzed, particularly for the history of flow and pressure of different flow fields during the inrush process. Therefore, it is important to build a coupled non-linear flow model considering the three flow fields: Darcy flow in the aquifer, high-velocity non-linear flow in the KCC and turbulent flow in the roadway during the entire flow process of water inrush.

In this study, in order to explain nonlinear flow behavior during the entire process of water inrush with better accuracy, the essence of flow transition was first verified by experiments. Then, a non-linear flow model coupling three flow fields was established based on the flow transition. The proposed model was finally used for numerical simulation of the process of water inrush in the Luotuoshan coal mine, China. In addition, the effect of the KCC permeability on flow behavior was discussed and some main conclusions were drawn.

## 2. Experiment on the flow translation of water inrush

Engineering practice shows that a water inrush disaster in a coal mine is the physical process of groundwater bursting from the aquifer into the mine roadway, which is induced by the combination of mining stress and high water pressure.^1,19,31^ The quantitative change of the inflow shows flow transitions from laminar flow in the aquifer to turbulent flow in the roadway. Zhao *et al.*^32^ postulated that water inrush from a bearing cave is induced by the failure of a rock pillar. After the failure of the rock pillar, water bursts out from the confined karst cave to form pipe flow. In physics, Darcy laminar flow is viscous resistance-based, and Navier–Stokes turbulent flow is inertial resistance-based. The state of Forchheimer flow is relatively complex and is based on both viscous resistance and inertial resistance. The classical theory of fluid mechanics uses the relationship between the Reynolds number and the Fanning friction coefficient to judge the transition between the above three states.^33^ In this section, a seepage experiment is conducted to study the flow transition and to provide an experimental basis for the establishment of the coupled non-linear flow model.

**Fig. 1:**
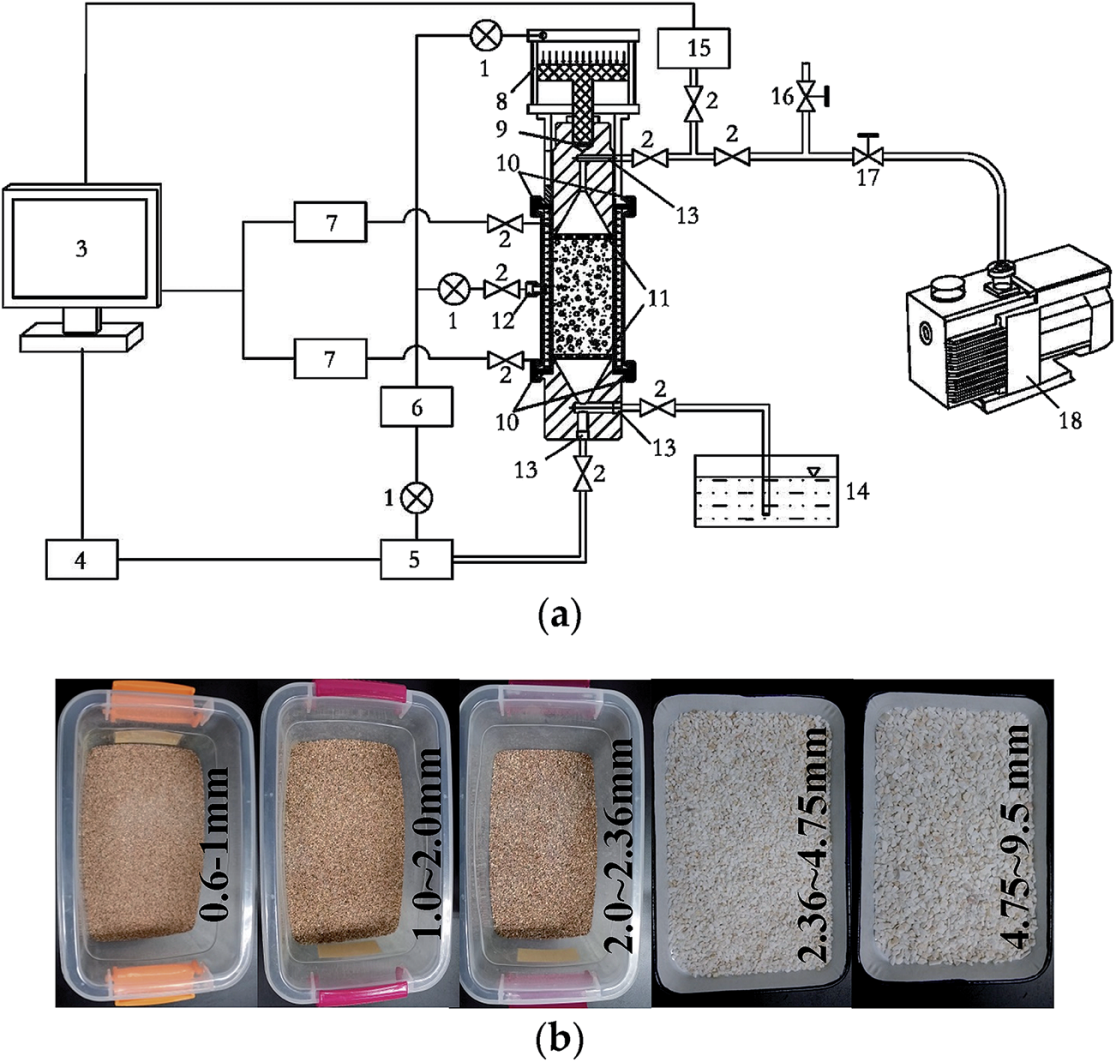
The testing system and sand samples: (a) schematic of the testing equipment of non-linear flow in porous media; (b) sand samples. (1) Pressure regulator; (2) stop valve; (3) computer; (4) servo motor; (5) water supply system; (6) air compressor (7) water pressure sensor; (8) air cylinder; (9) pluger; (10) clamp with locking; (11) perforated plate; (12) air intake; (13) inlet (outlet) of water; (14) water channel; (15) speed measuring device; (16) atmospheric valve; (17) pump out valve; (18) vacuum pump.

The testing system of non-linear flow in porous media, independently developed by Northeastern University, China, was employed to study the non-linear flow behavior of different porous media, as shown in Fig. 1a. The sample, 320 mm long and 60 mm in diameter, was saturated after being packed with sand. A constant water pressure was maintained during the experiment. The water entered from the bottom of the sample, and exited from the top of the sample. Piezometers were used to determine the pressure change along the column during the experiment and to calculate permeability. An electronic balance was used to measure water inflow. Data collection was carried out automatically by a computer with a 3 s sampling interval. The pre-set range of the hydraulic gradient was 3–36.5. Flow experiments were conducted on every sand specimen under 20 different water pressures by different grain diameter quartz sands (0.075–0.15, 0.15–0.3, 0.3–0.6, 0.6–1.0, 1.0–2.0, 2.0–2.36, 2.36–4.75, 4.75–9.5 mm) as shown in Fig. 1b. A thorough description of the device and experiment was presented by Yang *et al.*^34^

**Fig. 2:**
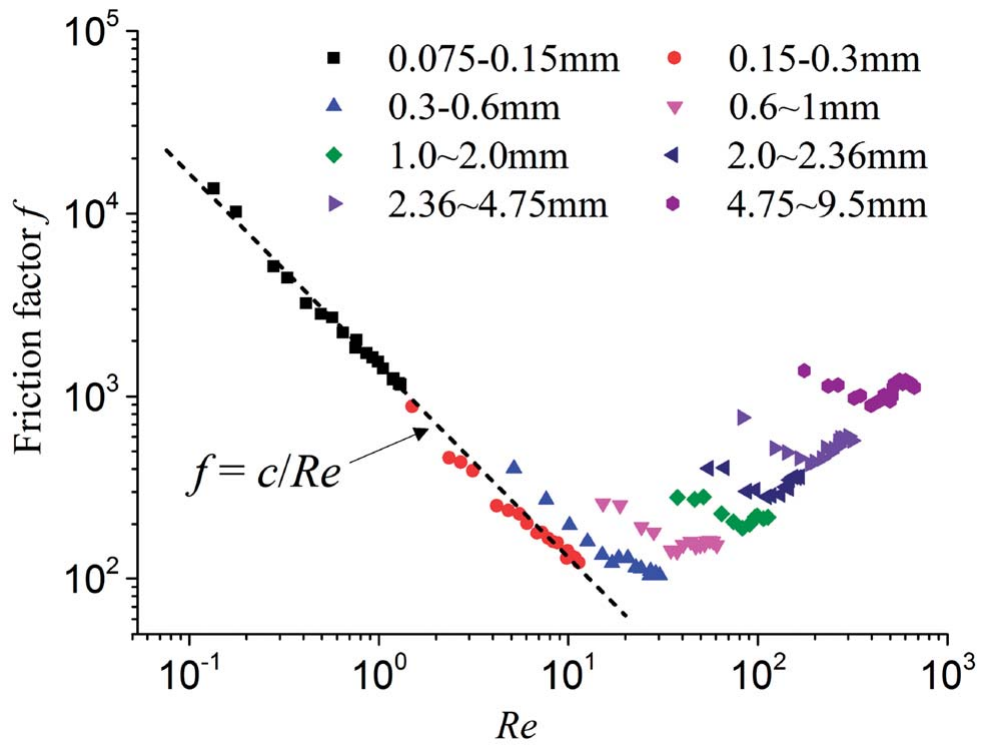
The relation of Reynolds number Re and Fanning friction coefficient *f* of different particle sizes.

Due to the inhomogeneity of the sands, the mean particle diameter was used as the characteristic length to calculate the Reynolds number and the Fanning friction coefficient.^33^ A relationship between the Reynolds number, Re, and the Fanning friction coefficient, *f*, was obtained and shown in Fig. 2. It can be observed that the flow in fine particle size sands satisfies Darcy's law. However, the relationship between Re and *f* gradually deviates from linearity with an increase in particle size, which indicates that the relationship deviates from Darcy's law and gradually transforms to Darcy–Forchheimer flow. When Re is significantly high, the Fanning friction coefficient does not decrease as the Reynolds number increases, but increases gradually. This indicates that the flow state is transitioning from laminar flow to turbulent flow, which has been confirmed by Tzelepis *et al.*^35^

In summary, under constant water pressure gradient, the flow state in porous media with different particle size sands is not necessarily the same. There are two flow transitions during the entire process of the experiments: (1) the transition from linear laminar flow to inertial flow; (2) the transition from inertial flow to turbulent flow. The larger the particle size, the more easily non-Darcy flow occurs. Therefore, for water inrush induced by KCC, the flow in the KCC may be in the intermediate state between laminar flow in the aquifer and turbulent flow in the roadway, and inevitably experiences flow transition from laminar flow to turbulent flow. This has also been verified through some studies by Yang *et al.*,^34^ Ma *et al.*,^6,7^ and Zhang *et al.*^36^ A flow model based on one flow state cannot reflect the flow transition of water inrush.

## 3. Model descriptions

### 3.1. Laminar flow in aquifer

Groundwater flow rate in an aquifer is usually small and the flow resistance is primarily viscosity dominated. The flow state of normal groundwater is laminar flow. The fluid velocity and pressure gradient satisfy the linear Darcy's Law,^33^ which can be expressed as1
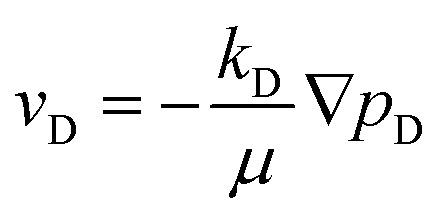
where *v*_D_ is the flow velocity (m s^−1^), *p*_D_ is the pressure (Pa), *k*_D_ is the permeability (m^2^), and *μ* is the dynamic viscosity (Pa s).

The continuity equation^33^ is2
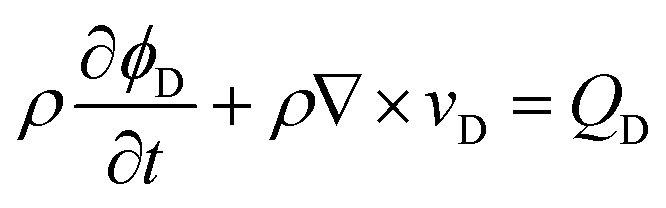
where *ρ* is the density of groundwater (kg m^−3^), *ϕ*_D_ is the porosity of aquifer, *Q*_D_ is the source (sink) term (kg m^−3^ s^−1^), and *t* is the time (s).

### 3.2. Forchheimer flow in KCC

The Forchheimer equation is widely used in the study of flow characteristics of earth and rockfill dams. Flow experiments conducted on fractured rocks indicate that the flow exhibits evident non-linear behavior and the relationship between the velocity and the pressure gradient effectively obeys the Forchheimer equation. Flow in fractured rocks is still laminar at a certain flow rate. The non-linear flow momentum equation of incompressible fluid in fractured rocks based on the Forchheimer equation^37^ can be written as3
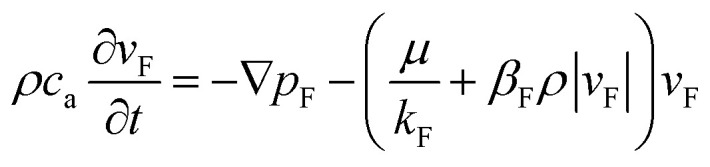


The continuity equation^33^ is given as4
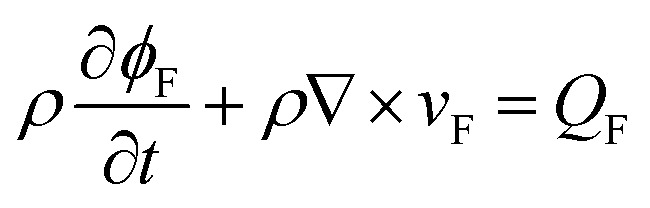


The relation between permeability and the non-Darcy factor can be given as^38,39^5
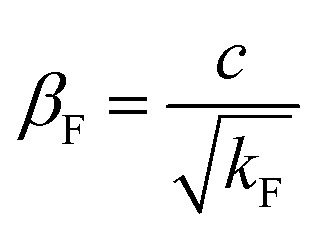
where *c*_a_ is the acceleration coefficient, *v*_F_ is the flow velocity (m s^−1^), *p*_F_ is the pressure (Pa), *k*_F_ is the permeability (m^2^), *β*_F_ is the non-Darcy factor (m^−1^), *c* is the Forchheimer coefficient, which can be obtained from seepage experiments, *Q*_F_ is the source (sink) term (kg m^−3^ s^−1^), and *ϕ*_F_ is the porosity of KCC.

### 3.3. Turbulent flow in roadway

The groundwater flow state in a mine roadway is turbulent flow, which satisfies the viscous Newtonian fluid Navier–Stokes equation,^40,41^ and can be expressed as6
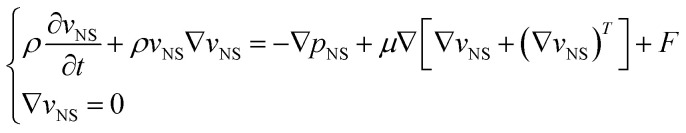
where *v*_NS_ is the flow velocity (m s^−1^), *p*_NS_ is the pressure (Pa), and *F* is the body force (N m^−3^).

### 3.4. Boundary conditions of the adjacent flow

During the entire process of groundwater travelling from the aquifer to the KCC and discharging into the roadway, both the flow rate and the pressure are continuous. Therefore, the transition boundary condition can be defined as follows:

On the boundary between the aquifer and the KCC7
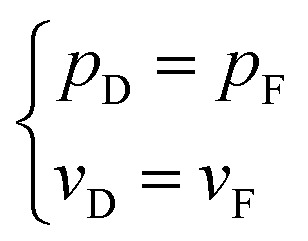


On the boundary between the KCC and the roadway8
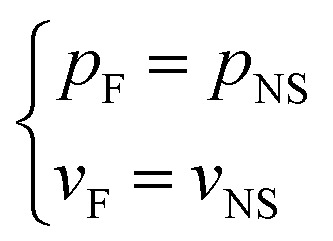


The above non-linear flow model is the combination of the flow in the aquifer, KCC, and roadway. This model can reflect the flow transitions from laminar flow in an aquifer to turbulent flow in a roadway.

### 3.5. Numerical methods and verification

The numerical solution of Darcy and Navier–Stokes is easy to implement and can be directly called in COMSOL. The nonlinear Forchheimer equation is solved by applying the Galerkin finite element method and simple linearization method.^42^ A more detailed analysis of numerical astringency while solving the equations has been presented in the literature.^27^ The finite element calculation program is compiled in Matlab, and the numerical solution of the coupled flow field can be realized through iterative solution using the module of COMSOL with Matlab.

A constant pressure is set for the inlet boundary of the aquifer, and a velocity, equaling the flow velocity of KCC at the interface, is set for the outlet boundary of the aquifer. As for the KCC, a pressure is set for the inlet boundary, which equals the pressure of the aquifer at the interface, and a velocity is set for the outlet boundary, which equals the flow velocity of the roadway at the interface. Furthermore, a pressure is set for the inlet boundary of the roadway, which equals the pressure of the KCC at the interface, and a constant pressure is set for the outlet boundary of the roadway. Specific details about the boundary conditions of the adjacent flow are provided in Table 1.

**Table 1:** Boundary conditions of the adjacent flow

Flow field	Boundary condition	
	Inlet	Outlet
Aquifer	*p*_1_ = const.	*v*_F_
KCC	*p*_D_	*v*_NS_
Roadway	*p*_F_	*p*_2_ = const.

**Fig. 3:**
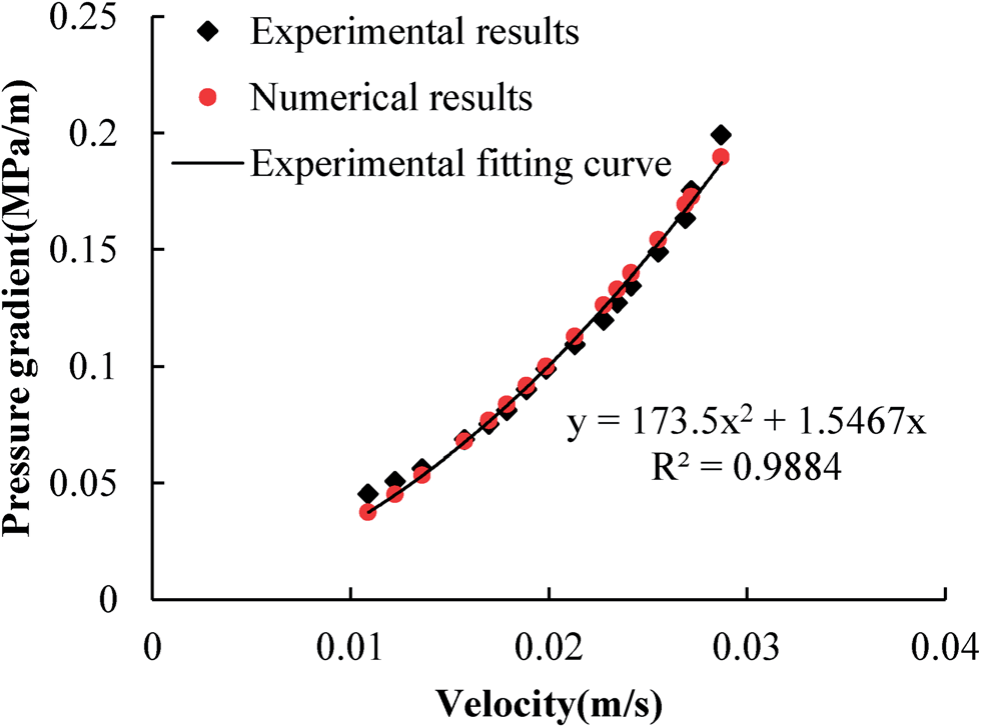
Comparison between laboratory results and numerical simulation using the Forchheimer equation.

As the built-in modules of COMSOL, the Darcy equation and the Navier–Stokes equation have been widely used. Therefore, only the finite element method for the Forchheimer equation needs to be verified. Fig. 3 shows the results of the seepage experiment of porous media with a particle size of 2.00–2.36 mm, from which the permeability, *k*_F_, and the non-Darcy factor, *β*_F_, of the Forchheimer equation can be obtained, *i.e.*, *k*_F_ = 6.465 × 10^−10^ m^2^ and *β*_F_ = 1.735 × 10^5^ m^2^. Based on the two medium parameters, the corresponding relationship between the pressure gradient and the flow velocity is obtained through numerical simulation. Fig. 3 shows that the numerical results are in good agreement with the experimental fitting results.

## 4. A case study

As a case study of water inrush from a coal seam floor, a numerical simulation based on the proposed non-linear flow model was conducted to investigate the flow behavior of the “3.1” water inrush incident in the Luotuoshan coal mine, China.

**Fig. 4:**
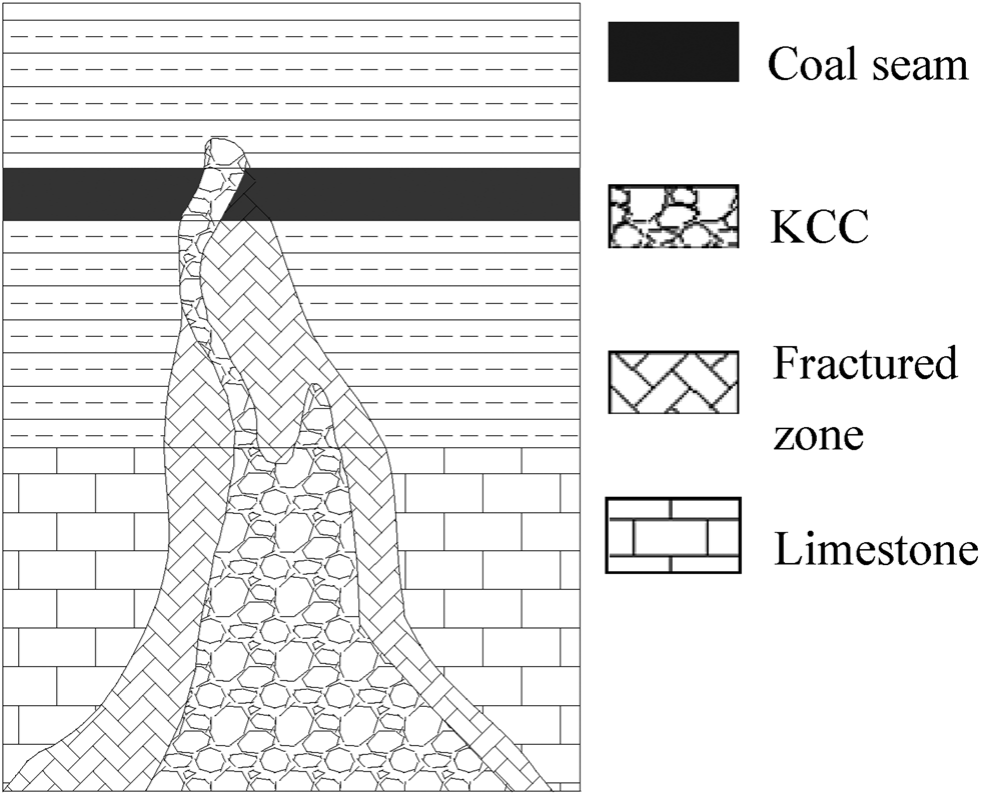
Speculated section of the KCC.

The water inrush occurred in the no. 16 coal seam on the +870 level of the Luotuoshan coal mine of the Shenhua Group on March 1, 2010. The maximum water inrush was up to 72 000 m^3^ h^−1^. In the incident, the mine was flooded, 32 miners died, and 7 miners were injured. The direct economic loss was about RMB 48 million. A hydrogeological investigation and a pumping test indicated that the water inrush source was the Ordovician limestone karst aquifer with abundant water content. The average thickness of the aquifer is about 23 m, and the Ordovician aquifer water pressure is about 4.1 MPa. Hydrogeological investigation and similar material model tests indicate that the water-conducting channel is a developing KCC. The speculated section of the KCC is shown in Fig. 4. The area of the excavated cross section of the no. 16 coal seam is 19 m^2^. The water inrush occurred at the working face of the coal seam roadway.

### 4.1. Numerical model

**Fig. 5:**
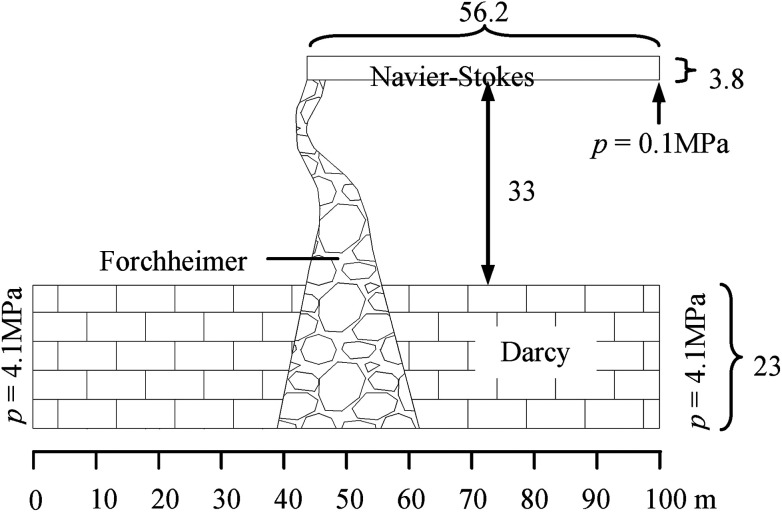
Numerical model of water inrush induced by the KCC in the Luotuoshan coal mine.

According to the geologic and geometric structure of the water inrush induced by the KCC in the Luotuoshan coal mine, a two dimensional numerical model is established as shown in Fig. 5. The model comprises three parts: the limestone aquifer with an area of 100 m × 23 m, the water-conducting channel of the KCC with a maximum width of 56 m, height of 21.5 m, and the roadway with an area of 56.2 m × 3.8 m. According to the hydrological conditions, the boundary conditions of the model were set as follows. A constant hydraulic pressure, *p* = 4.1 MPa, was set for the left and right boundaries of the aquifer. Constant atmospheric pressure, *p* = 0.1 MPa, was set for the right exit of the roadway. At the interface of the aquifer and the KCC, *p*_D_ = *p*_F_ and *v*_D_ = *v*_F_ were set. At the interface of the KCC and the roadway, *p*_F_ = *p*_NS_ and *v*_F_ = *v*_NS_ were used. A boundary condition with zero flux was applied at the rest of the external boundary of the model. An initial water pressure, *p* = 4.1 MPa, was set for the aquifer and the KCC. According to the field test, the hydrodynamic parameters are shown in Table 2.

**Table 2:** Hydraulic parameters for the simulations

Flow field	Aquifer	KCC	Roadway
Flow regime	Darcy flow	Forchheimer flow	Turbulent flow
Density (kg m^−3^)	1000	1000	1000
Viscosity (Pa s)	0.001	0.001	0.001
Porosity *φ*	0.14	0.348	—
Permeability *k* (m^2^)	2.1 × 10^−11^	*k*_F_	
Acceleration coefficient *c*_a_	—	1.0	
Forchheimer coefficient		9.8	

**Fig. 6:**
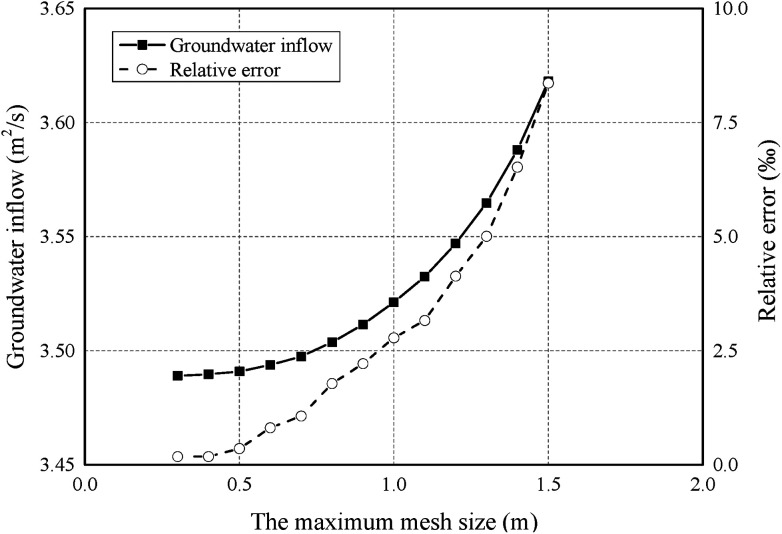
The effect of mesh size on groundwater inflow and relative error.

The accuracy of the finite element calculation is largely related to the quality of the finite element mesh. In general, the smaller the mesh size, the more accurate the calculation result and the more time-consuming the calculation. Thus, an error analysis was first conducted to determine the most suitable mesh size in order to eliminate the effect of the finite element mesh on the calculation results as much as possible. Fig. 6 shows the results of groundwater inflow and relative error under different maximum mesh size using *k*_F_ = 9.6 × 10^−9^ m^2^. As shown in Fig. 6, the calculation error increases with the maximum mesh size. Considering the calculation efficiency and the calculation error, the model is discretised into a mesh that contains 19 370 six-node triangle elements with a maximum mesh size of 0.6 m and a relative error of 1%. A time step of 2 s was set for the models and the total time was 180 s.

### 4.2. Numerical results

**Fig. 7:**
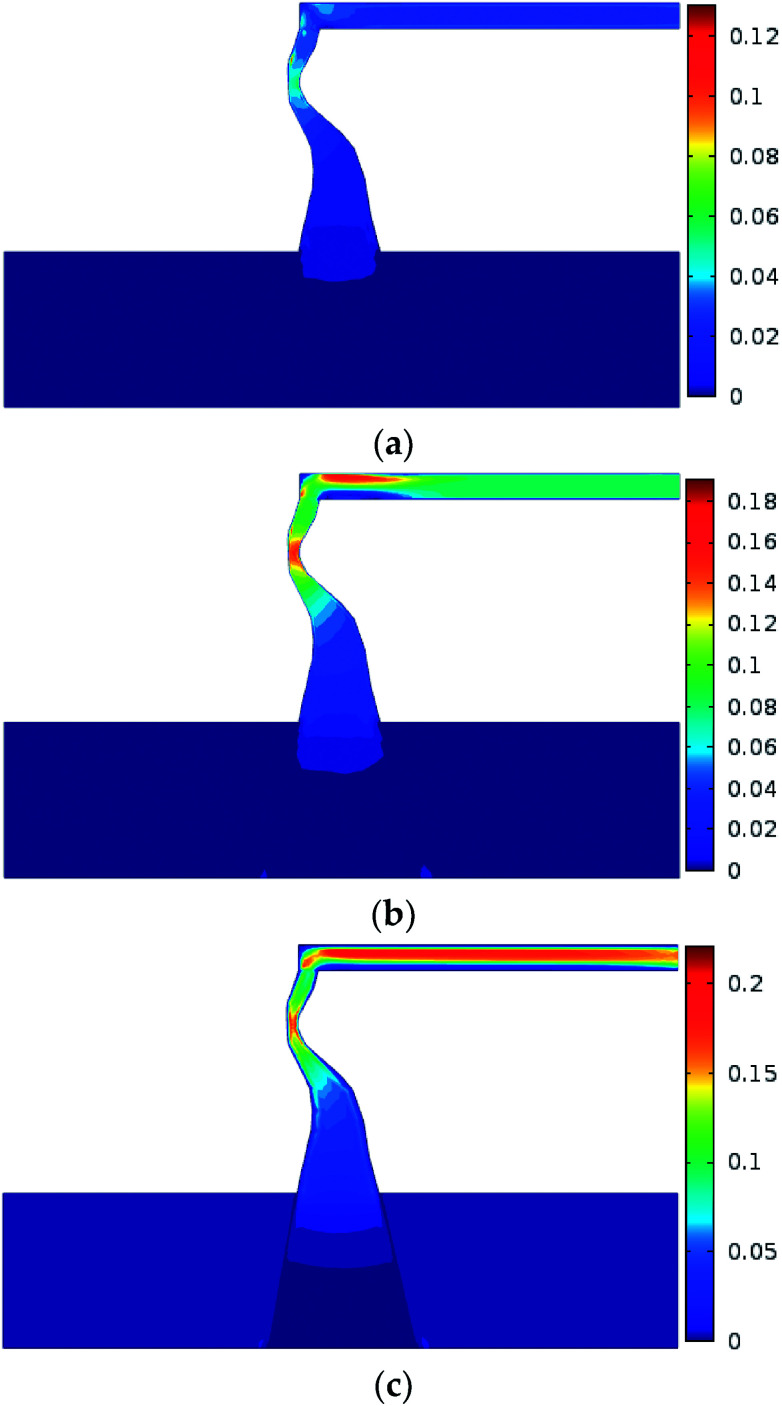
Distribution of the flow velocity during the water inrush process: (a) *t* = 1 s; (b) *t* = 60 s; (c) *t* = 180 s (unit: m s^−1^).

**Fig. 8:**
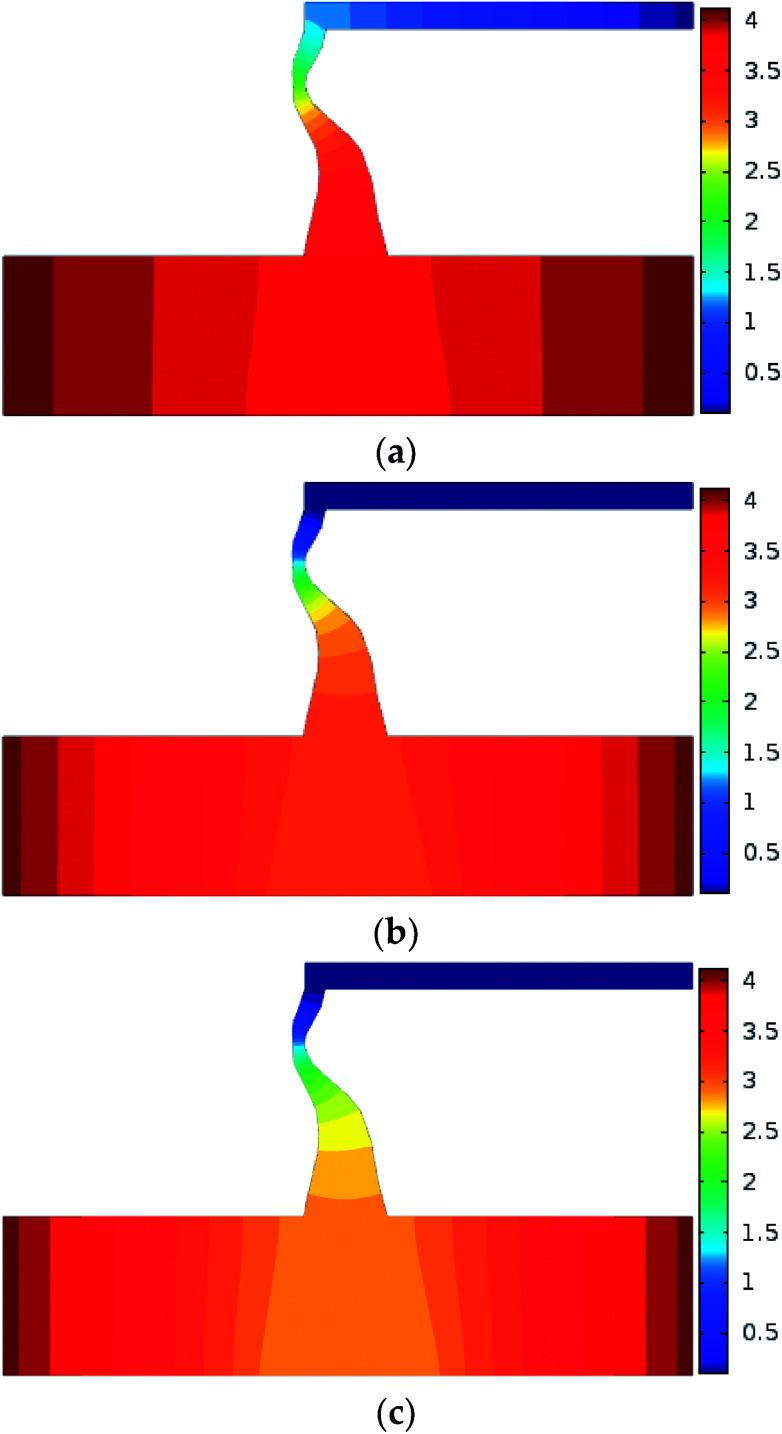
Distribution of the flow pressure during the water inrush process: (a) *t* = 1 s; (b) *t* = 60 s; (c) *t* = 180 s (unit: MPa).

**Fig. 9:**
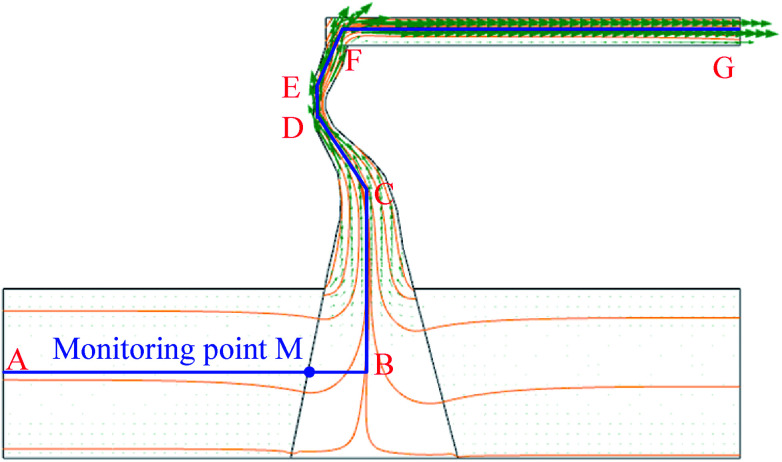
Distribution of the streamline and flow velocity.

**Fig. 10:**
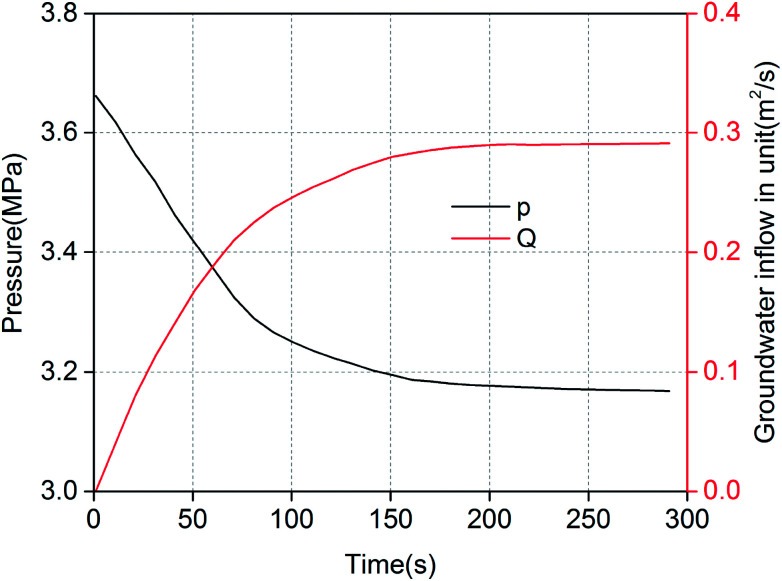
Water inflow and pressure history at the inlet of the KCC.

Numerical simulation of the water inrush was conducted using *k*_F_ = 9.6 × 10^−9^ m^2^. The results are shown in Fig. 7–10. Fig. 7 and 8 represent the distributions of the water velocity and pressure. Fig. 9 shows the distribution of the streamlines and the velocity vectors. The water inflow history of the measuring point *M* is shown in Fig. 10. As shown in Fig. 7 and 8, the flow velocity and pressure changes continuously during the entire process. The flow state of the inrush in the KCC is Forchheimer flow, between Darcy laminar flow dominated by the viscous resistance and turbulent flow dominated by the inertial resistance. The flow velocity increases with time. Forchheimer flow is first dominated by viscous resistance. Gradually, it is dominated by both inertial resistance and viscous resistance. Finally, Forchheimer flow is completely dominated by the inertial resistance. Therefore, the water inrush induced by a KCC is a dynamic process of flow changing from gradual to sudden. As shown in Fig. 10, water flow with high pressure in the KCC rushes into the roadway continuously as the fluid pressure in the KCC is translated into momentum of the fluid. Therefore, the pressure on the entrance of the KCC drops gradually; conversely, the water inflow increases gradually. As the confined aquifer boundary pressures remain constant, the pressure distribution in the KCC and aquifer will eventually attain equilibrium according to the pressure balance principle. In addition, the computed water flow at the outlet of the roadway will reach a steady state.

### 4.3. Sensitivity analysis of the KCC permeability

During the water inrush process induced by KCC, the KCC permeability constantly increases, due to the loss of sediment and broken stone particles under the effects of high pressure and high rate flow scouring.^7,19^ Under certain water pressure conditions, the KCC permeability and the non-Darcy factor not only directly affect the relative weights of the viscous resistance and inertial resistance, but also decide the water pressure distribution in the KCC and the water inflow in the roadway. There is a one-to-one correspondence between the permeability and non-Darcy factor.^21,22^ Therefore, sensitivity analysis of the KCC permeability has implications for the elucidation of the mechanism of water inrush.

#### 4.3.1. The method of discriminating the flow state

The Forchheimer number is the ratio of the quadratic term of the Forchheimer equation (the inertial term) and the first-degree term (viscosity term), which could reflect the non-linear degree of the non-linear flow. It was applied in discriminating non-linear flow by Ruth and Ma^43^ and Zeng and Grigg:^44^9
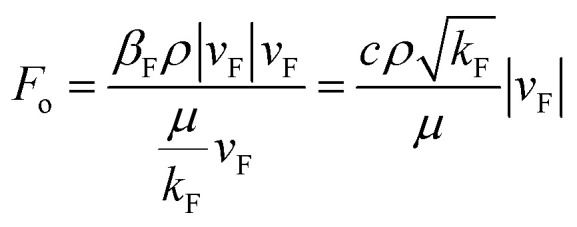


Eqn (9) shows that when the flow velocity is sufficiently small (*F*_o_ ≪ 1), the flow inertial resistance can be ignored compared with the viscous resistance. The flow state tends to be Darcy laminar, which shows weak nonlinearity. When the flow velocity is high enough (*F*_o_ ≫ 1), the flow inertial resistance cannot be ignored compared with the viscous resistance. The flow state tends to be turbulent, which shows strong nonlinearity. When the velocity is in a certain range (*F*_o_ ≈ 1), the inertial resistance is about the same as the viscous resistance. Neither of the resistances can be ignored, and the flow state is non-linear laminar controlled by both the inertial resistance and viscous resistance.

#### 4.3.2. The effect of permeability on the flow state

**Fig. 11:**
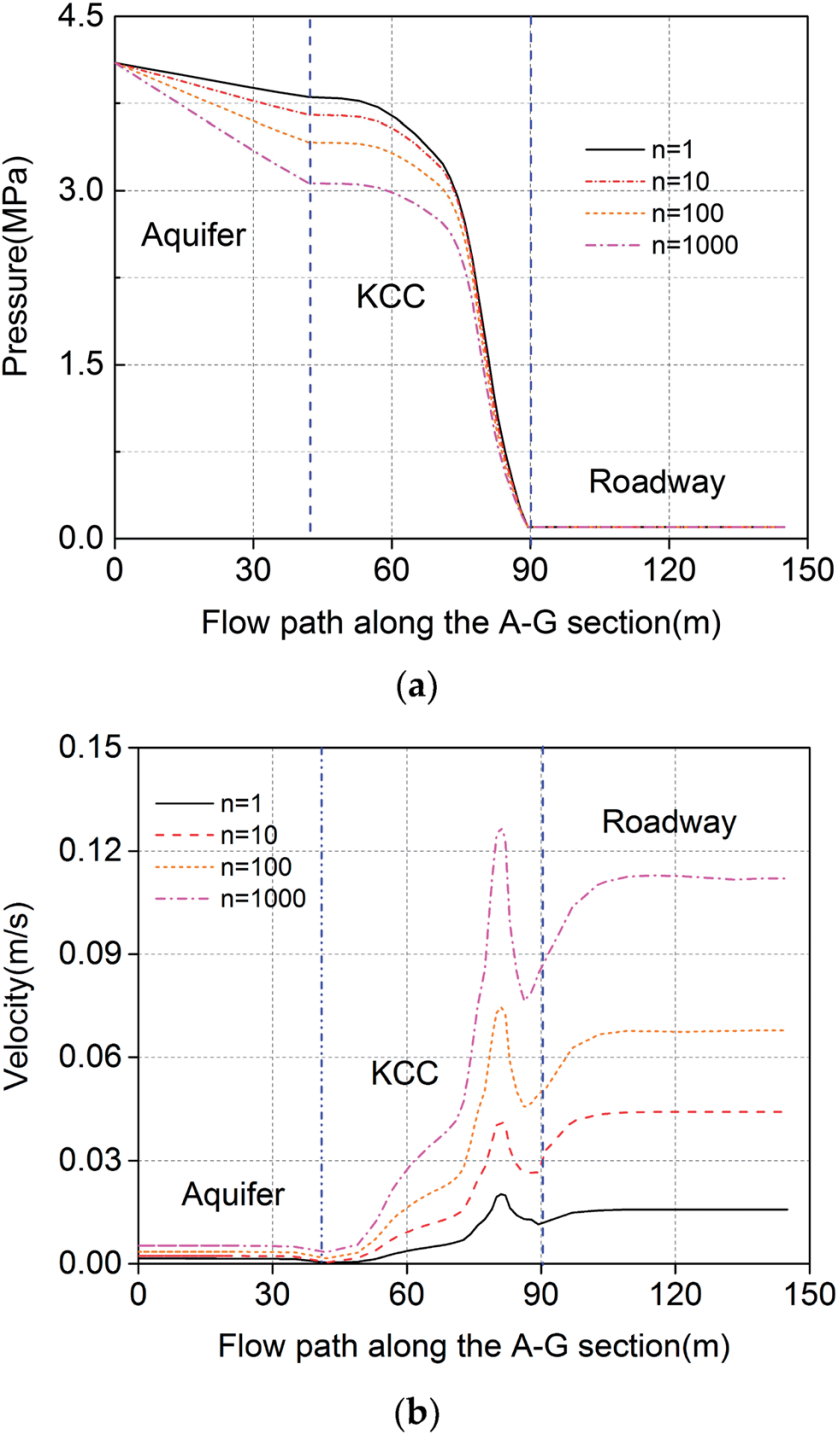
Water pressure and velocity along the flow path under different ratios of permeability: (a) pressure; (b) velocity.

In order to study the effect of the KCC permeability on the flow state, a variable *n* was defined as the ratio of the KCC permeability to the aquifer permeability, which can be expressed as *n* = *k*_F_/*k*_D_. There is a one-to-one correspondence between each *n* and the permeability; the greater the value of *n*, the higher the KCC permeability. In this study, the variable *n* is set in a wide range (10^0^–10^3^). For each magnitude of *n*, the water pressure and velocity on the flow path when the flow is stable is extracted and illustrated in Fig. 11. It can be observed that the fluid pressure and velocity in the KCC are very sensitive to the KCC permeability. The permeability affects the non-Darcy effect and thus, affects the non-linear inertial resistance. Under the constant aquifer pressure boundaries, the higher the KCC permeability, the greater is the pressure drop in the aquifer. The flow velocity at the same location in the KCC increases as the KCC permeability increases. Because the KCC is shaped like a plug (the width of the bottom is obviously bigger than the top), according to the mass conservation of fluids, the narrower the KCC channel, the greater is the flow velocity. Taking *n* = 10^3^ as an example, the highest velocity in the channel can be up to 0.13 m s^−1^, and is located in the narrowest part of the channel.

**Fig. 12:**
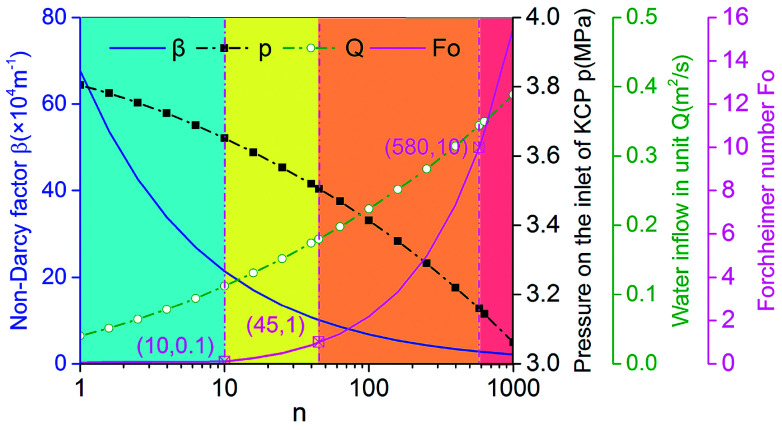
Permeability sensitivity analysis.

Fig. 12 shows the relationship between *n* and the non-Darcy factor, Forchheimer number, water inflow and water pressures at the entrance of the KCC (measuring point *M*). It can be observed that, with an increase in permeability, the non-Darcy factor and the pressure at the entrance of the KCC decreases, while the Forchheimer number and the water inflow increases gradually. When the variable *n* ranges from 10^0^ to 10^3^, the Forchheimer number changes from 10^−2^ to 10^1^. According to the Forchheimer number and the variable *n*, the curve can be divided into three phases:

(1) *n* < 10: the Forchheimer number is smaller than 0.1, which indicates that the inertial resistance is smaller than 10% of the viscous resistance. The effect of the inertial resistance on the flow velocity is weak. The flow in the KCC tends to be Darcy flow.

(2) 10 < *n* < 580: the Forchheimer number is in the range of 0.1–10, the fluid velocity is controlled by both viscous resistance and inertial resistance. However, as the KCC permeability increases, the flow in the KCC is initially dominated by viscous resistance, and then dominated by inertial resistance. When *n* = 45, the Forchheimer number is about 1, the viscous resistance and the inertial resistance are approximately the same. This phase can be further divided as 10 < *n* < 45 for weak inertial flow and 45 < *n* < 580 for strong inertial flow.

(3) *n* > 580: the Forchheimer number is higher than 10; thus, the inertial resistance in the KCC is at least 10 times that of the viscous resistance. The viscous resistance weakly affects the flow velocity. The fluid flow in the KCC is non-linear laminar flow, which tends to be turbulent flow.

The results further illustrate that the Forchheimer equation applied to describe the high non-linear laminar flow can reflect the intermediate state of the flow translation from the laminar flow in an aquifer to turbulent flow in a roadway, and also can reveal the essence of the three flow transition in the process of water inrush. When the water pressure in the aquifer is constant, the KCC permeability is an important indicator of transition of the fluid flow state in the KCC.

### 4.4. Further discussion

As mentioned above, when the velocity is sufficiently low, the Forchheimer equation can be approximately replaced by the Darcy equation. In other words, the Darcy equation is a particular case of the Forchheimer equation at low velocity. Assuming that the flow in the aquifer can be described by the Forchheimer equation, the critical Forchheimer number, *F*_o_ = 0.1, is capable of identifying the beginning of non-Darcy flow.^44^ Hence, with the increase of the KCC permeability, the flow in the aquifer does not exactly satisfy Darcy's law and the flow in the KCC is also not entirely non-Darcy.

**Fig. 13:**
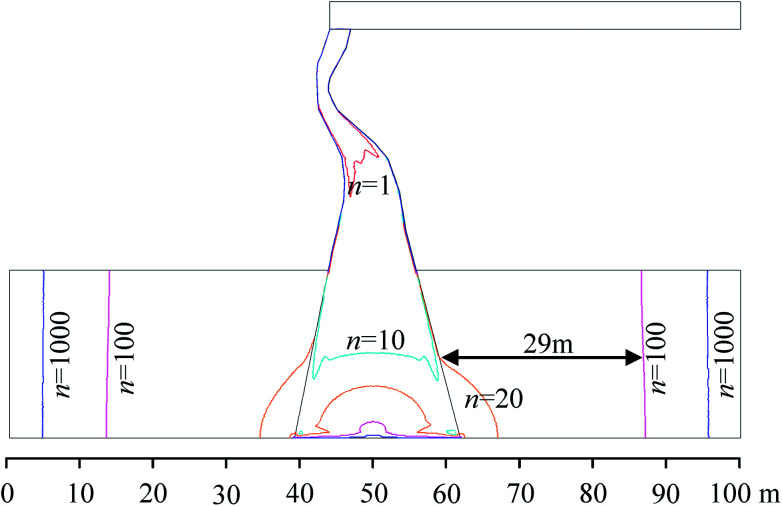
Changes in the non-Darcy effect region with different variable *n*.

Fig. 13 shows the distribution of the boundaries of the non-Darcy effect region under different permeability variation coefficients, *n*. It can be observed that for flow in the KCC, when *n* = 1, non-Darcy flow occurred in the top narrow part of the KCC first, while Darcy flow occurs at the lower wide part. With the KCC permeability increasing, non-Darcy flow expanded to the lower part and the entire KCC is almost non-Darcy flow till *n* = 100. For flow in the aquifer, when *n* = 20, there is a small non-Darcy flow region in the aquifer near the KCC. Roughly speaking, the flow in the aquifer satisfies Darcy's law basically if *n* < 20. With increasing *n*, the non-Darcy flow region in the aquifer will extend away from the KCC rapidly. When *n* increases to 100, the boundary of non-Darcy flow region in the aquifer is about 26 m away from the KCC. With further increase of *n*, the non-Darcy flow region will grow slowly. This physical process is similar to the spread of drawdown due to a pumping well in hydraulic engineering.^25,26^

Overall, during the entire process of water inrush induced by KCC, the flow behavior, both in the aquifer and the KCC, may present Darcy and non-Darcy flow under high water pressure. This primarily depends on the KCC permeability. The higher the KCC permeability, the more significant is the non-Darcy behavior and hence, the higher is the risk of water inrush. In engineering practice, the proper flow model should be selected according to the hydraulic conductivity of KCC and the study region of the aquifer to enable a contribution towards a reasonable prediction of water inflow and an assessment of potential water inrush. If the study region of the aquifer is wide or the permeability of the KCC is low, the non-Darcy region in the aquifer can be ignored. If the local region of the aquifer is the main study area and the KCC permeability is high, the non-Darcy model can be used to describe the seepage in the aquifer. The discussion can also provide a reference for further establishing a finer model, *e.g.*, the aquifer has a non-Darcy flow boundary.^26^

## 5. Conclusions

A non-linear flow model was established to simulate the process of water inrush induced by KCC and was used to study the flow behavior of inrush in the Luotuoshan coal mine. The main conclusions can be drawn as follows:

(1) To more accurately reflect the nature of water inrush induced by KCC in the coal seam floor, a non-linear flow model that couples three fields, *i.e.*, Darcy flow in the aquifer, high-velocity non-linear flow in the KCC and turbulent flow in the roadway, is established. The proposed model reflects the essence of transition of flow states during the entire dynamic process of water inrush induced by KCC.

(2) The flow state of the inrush water in the KCC is a crossbreed between Darcy laminar flow, dominated by viscous resistance, and Forchheimer flow, dominated by inertial resistance. The flow velocity increased with time and the Forchheimer flow is initially dominated by viscous resistance, then dominated by both inertial resistance and viscous resistance, and finally dominated by inertial resistance. Therefore, the water inrush induced by KCC is a dynamic flow process with the evolution of flow types.

(3) During water inrush induced by KCC, the filled sediments and broken stone particles in KCC are washed away under the effects of high pressure and high flow rate scouring. Consequently, the permeability of the KCC evidently increases. This further indicates that the Forchheimer equation can reflect the flow characteristics over a wide range of permeabilities and flow rates, and can also reflect the intermediate state of the flow translation from laminar flow in the aquifer to turbulent flow in the roadway during the water inrush process.

## Author contributions

Tianhong Yang, Xiangang Hou and Wenhao Shi contributed to the formulation of overarching research goals and aims; Xiangang Hou and Wenhao Shi analyzed the data; Wenhao Shi and Xiangang Hou wrote the paper; Wenhao Shi and Xiangang Hou contributed to the numerical calculations and simulations.

## Conflicts of interest

The authors declare no conflicts of interest.

## Supplementary Material

## References

[cit1] Yin S. X., Zhang J. C. (2005). Environ. Geol..

[cit2] Zhang J. C., Shen B. H. (2004). Int. J. Rock Mech. Min. Sci..

[cit3] Li G. Y., Zhou W. F. (2005). Environ. Geol..

[cit4] Zhong Y. P. , Comprehensive technology research on water control of Kailuan mines, Coal Industry publishing house, Beijing, 2001

[cit5] Liu Z. W. , Karst Waterburst mechanism and prevention countermeasures in Yuanliangshan tunnel, China University of Geosciences, 2004

[cit6] Ma D., Miao X. X., Jiang G. H., Bai H. B., Chen Z. Q. (2014). Transp. Porous Media.

[cit7] Ma D., Bai H. B., Miao X. X., Pu H., Jiang B. Y., Chen Z. Q. (2016). Environ. Earth Sci..

[cit8] Ma D., Rezania M., Yu H. S., Bai H. B. (2016). Eng. Geol..

[cit9] Ma D., Miao X. X., Bai H. B., Pu H., Chen Z. Q., Liu J. F., Huang Y. H., Zhang G. M., Zhang Q. (2016). Environ. Earth Sci..

[cit10] Shi W. H., Yang T. H., Liu H. L., Yang B. (2018). J. Hydrol. Eng..

[cit11] Li B. J., Garga V. K., Davies M. H. (1998). J. Hydraul. Eng..

[cit12] Wen Z., Wu F. X., Feng Q. G. (2016). J. Hydrol. Eng..

[cit13] Chen Z. X., Lyons S. L., Guan Q. (2001). Transp. Porous Media.

[cit14] Panfilov M., Fourar M. (2006). Adv. Water Resour..

[cit15] Pradip K. G. N., Venkataraman P. (1995). J. Inst. Eng. (India), Civ. Eng. Div..

[cit16] Sidiropoulou M. G., Moutsopoulos K. N., Tsihrintzis V. A. (2007). Hydrol. Processes.

[cit17] Li J., Huang G. H., Wen Z., Zhan H. B. (2008). J. Hydraul. Eng..

[cit18] Cherubini C., Giasi C. I., Pastore N. (2012). Hydrol. Earth Syst. Sci. Dis..

[cit19] Miao X. X., Li S. C., Chen Z. Q., Liu W. Q. (2011). Transp. Porous Media.

[cit20] Sedghi-Asl M., Rahimi H., Salehi R. (2014). Transp. Porous Media.

[cit21] Moutsopoulos K. N., Papaspyros I. N. E., Tsihrintzis V. A. (2009). J. Hydrol..

[cit22] Moutsopoulos K. N., Tsihrintzis V. A. (2005). J. Hydrol..

[cit23] Hansen D., Garga V. K., Townsend D. R. (1995). Can. Geotech. J..

[cit24] Panthulu T. V., Krishnaiah C., Shirke J. M. (2001). Eng. Geol..

[cit25] Basak P. (1977). J. Hydrol..

[cit26] Wang Q., Zhan H., Tang Z. (2014). Hydrol. Earth Syst. Sci. Dis..

[cit27] Xu Z. H., Li S. C., Li L. P., Shi S. S. (2011). Adv. Mater. Res..

[cit28] Xu Z. H., Li S. C., Li L. P., Shi S. S. (2011). Adv. Mater. Res..

[cit29] Yang T. H., Shi W. H., Li S. C., Yang X., Yang B. (2016). J. China Coal Soc..

[cit30] Irmay S. (1958). Eos, Trans., Am. Geophys. Union.

[cit31] Shi W. H., Yang T. H., Yu Q. L., Li Y., Liu H. L., Zhao Y. C. (2017). Mine Water Environ..

[cit32] Zhao Y. L., Zhang S. G., Wan W., Wang W. J., Cai L., Peng Q. Y. (2014). Chin. J. Rock Mech. Eng..

[cit33] Bear J. , Dynamics of fluids in porous media, Dover Publications, New York, 1972

[cit34] Yang X., Yang T. H., Xu Z. H., Yang B. (2017). Energies.

[cit35] Tzelepis V., Moutsopoulos K. N., Papaspyros J. N. E., Tsihrintzis V. A. (2015). J. Hydrol..

[cit36] Zhang B. Y., Bai H. B., Zhang K. (2016). Rock Soil Mech..

[cit37] Ni X. Y., Kulatilake P. H. S. W., Chen Z. Q., Gong P., Kong H. L. (2016). Geotech. Geol. Eng..

[cit38] Dukhan N., Bağcı Ö., Özdemir M. (2014). Exp. Therm. Fluid Sci..

[cit39] Bağcı Ö., Dukhan N., Özdemir M. (2014). Transp. Porous Media.

[cit40] Foias C. , Manley O., Rosa R. and Temam R., Navier-Stokes equations and turbulence, Cambridge University Press, Cambridge, 2001

[cit41] Li S. C., Xu Z. H., Ma G. W. (2014). Tunn. Undergr. Space Technol..

[cit42] Zhang J., Xing H. L. (2012). Geothermics.

[cit43] Ruth D., Ma H. P. (1992). Transp. Porous Media.

[cit44] Zeng Z. W., Grigg R. (2006). Transp. Porous Media.

